# Exploring Ozonated Vegetable Oils as Antimicrobial and Functional Agents in Food Systems: A Systematic Narrative Review

**DOI:** 10.3390/foods15111850

**Published:** 2026-05-23

**Authors:** Paula Dominguez-Lacueva, Paula Corella-Guillamón, María J. Cantalejo-Díez

**Affiliations:** Institute for Sustainability & Food Chain Innovation (IS-FOOD), Jerónimo de Ayanz Building, Arrosadia Campus, Public University of Navarre (UPNA), E-31006 Pamplona, Spain; paula.dominguez@unavarra.es (P.D.-L.); paula.corella@unavarra.es (P.C.-G.)

**Keywords:** ozone, food preservation, vegetable oils, antimicrobial activity, antioxidant activity, packaging, functionality

## Abstract

Ozonated vegetable oils are increasingly recognized as bioactive agents with both antimicrobial and functional properties, attracting growing interest for their potential applications in food systems. This review critically synthesizes current knowledge on their chemical transformations, physicochemical properties, antimicrobial and functional properties, their toxicological and safety considerations and the implications of their regulatory classification based on 84 studies published between 2005 and 2026. Evidence shows that antimicrobial performance increases with oxidation level, but significant variability in ozonation conditions and analytical methods limits comparability and hinders standardization. Despite encouraging in vitro results and early applications in food matrices and packaging systems, major gaps remain regarding toxicology, sensory quality, regulatory classification, and real-world functionality. Overall, ozonated vegetable oils hold notable potential as antimicrobial and functional tools, yet further research is required to ensure their safe and practical implementation in food systems.

## 1. Introduction

Food preservation remains one of the most critical challenges facing the global food industry in the 21st century. The World Health Organization [1] estimates that approximately 600 million people—almost 1 in 10 individuals worldwide—fall ill after consuming contaminated food each year, resulting in 420,000 deaths and the loss of 33 million healthy life years (DALYs). The economic burden of foodborne diseases in low- and middle-income countries alone exceeds $110 billion annually, underscoring the profound public health and economic implications of inadequate food safety measures. As global food supply chains become increasingly complex and consumers’ demand for minimally processed, preservative-free products intensifies, the food industry faces intense pressure to develop novel preservation technologies that can ensure microbiological safety while maintaining nutritional quality, sensory attributes, and clean-label appeal [2].

Traditional preservation methods—thermal processing, chemical preservatives, refrigeration, and modified atmosphere packaging—have served the industry well for decades but are increasingly perceived as incompatible with the current complexity, sustainability demands, and cost-efficiency requirements of the modern food industry [3]. Synthetic chemical preservatives such as benzoates, sorbates, and nitrites, while effective, face growing regulatory scrutiny and consumer rejection due to concerns about potential health effects [4] and their association with highly processed foods. This paradigm shift has catalyzed intensive research into natural antimicrobial agents derived from plants, essential oils, organic acids, bacteriocins, and other biological sources.

Among emerging preservation technologies, ozone-based approaches have gained significant attention due to ozone’s powerful oxidizing properties, broad-spectrum antimicrobial activity, and decomposition into non-toxic oxygen without leaving chemical residues [5,6,7]. The use of ozone in the food industry has expanded significantly since the U.S. Food and Drug Administration granted it Generally Recognized as Safe (GRAS) status for use as an antimicrobial agent in food processing in 2001 [8]. Ozone applications in food systems encompass multiple domains: gaseous ozone for surface decontamination of fresh produce like beef [9], pork [10] or turkey meat [11]; aqueous ozone for washing fruits and vegetables [12] and food contact surfaces [13]; and ozone-enriched atmospheres for storage of perishable commodities [14].

Ozone’s efficacy in reducing microbial loads on various food products has been proven by extending shelf life and controlling foodborne pathogens, including bacterial pathogens (*Salmonella* spp., *Listeria monocytogenes*, *Escherichia coli* or *Staphylococcus aureus*), spoilage microorganisms (lactic acid bacteria, *Pseudomonas* spp., *Enterobacteriaceae*), and fungal contaminants (*Aspergillus*, *Alternaria*, *Penicillium* species) that produce mycotoxins [15].

However, the application of gaseous ozone in food systems is constrained by its instability, short half-life, limited penetration into food matrices, and potential for inducing undesirable oxidative changes in lipids and other sensitive food components. Ozone typically has a 20–30 min of half-life in aqueous solutions at room temperature [16] and shows a rapid decomposition in the presence of organic matter, limiting its residual antimicrobial activity. The gas has limited penetration into porous food matrices, crevices, and biofilms, reducing its effectiveness against protected microbial populations. Moreover, high ozone concentrations or prolonged exposure can induce undesirable oxidative changes in food components, including lipid oxidation, protein denaturation, color changes, and off-flavor development [17].

These limitations have prompted researchers to explore alternative delivery systems for ozone’s antimicrobial properties, leading to the development of ozonated vegetable oils that have been chemically modified through controlled ozonation to incorporate stable oxygenated compounds with antimicrobial activity [18]. Different vegetable oil types (sunflower, olive, coconut, hemp, krill…) have been used for ozonation purposes. Ozonated vegetable oils represent a distinct class of antimicrobial agents produced through the controlled reaction of ozone gas with the unsaturated fatty acids present in vegetable oils. The ozonation process transforms the chemical structure of the oil through ozonolysis reactions, generating a complex mixture of oxygenated compounds, including ozonides, peroxides, aldehydes, ketones, and carboxylic acids. These oxygenated species are responsible for the antimicrobial activity of ozonated oils, with ozonides—cyclic peroxides formed through the addition of ozone across carbon-carbon double bonds—proposed as key active agents [19]. The quality of the ozonation process of oils depends on different factors such as O_3_ concentration, ozonation time, temperature and the composition of the selected olive oil [20,21]. Unlike gaseous or aqueous ozone, ozonated oils exhibit remarkable stability, with antimicrobial activity reportedly maintained for months or years when stored under appropriate conditions, making them practical for commercial food applications [22]. In addition, they have attracted increasing attention not only as stable antimicrobial systems for food applications, but also as bioactive functional agents, due to their reported antioxidant, anti-inflammatory, tissue-protective, and wound-healing properties in different experimental models [19].

The rationale for ozonated vegetable oils as an antimicrobial agent in food systems rests on several key attributes: (1) broad-spectrum antimicrobial activity against bacteria, fungi, and biofilms; (2) stability of antimicrobial compounds over extended storage periods; (3) natural origin and consumer acceptance of vegetable oils as a food ingredient; (4) compatibility with food matrices and packaging materials; (5) potential for controlled release through encapsulation technologies; (6) absence of chemical residues or synthetic additives, and (7) evidence of additional functional properties, including antioxidant and anti-inflammatory activities. However, the translation of these potential advantages into practical food applications requires rigorous evaluation of efficacy, safety, stability, sensory impact, and regulatory compliance—areas where significant knowledge gaps currently exist.

In this context, the objective of this review is to provide an overview of ozonated vegetable oils as emerging antimicrobial and functional agents in food systems. The review focuses on elucidating their chemical nature, production processes, and physicochemical characterization, with particular emphasis on analytical and technological approaches used to monitor ozonation. Furthermore, it examines their antimicrobial, antioxidant and anti-inflammatory activities, as well as the key aspects related to sensory impacts, safety, toxicological considerations, and current regulatory frameworks. By integrating these dimensions, this work aims to clarify the real potential and limitations of ozonated oils for their effective and responsible use in modern food systems.

## 2. Methodology

This study was designed as a systematic narrative review. The systematic component was ensured by following the recommended guidelines for Preferred Reporting Items for Systematic Reviews and Meta-Analyses (PRISMA) and was used for literature identification, screening and study selection [23]. In this regard, a search strategy was conducted to retrieve the available research works about ozonated vegetable oils related to food sciences or the food industry. The search was conducted in two international databases covering the last twenty years, from 2005 to 2026: Web of Science and Scopus. The search consisted of a combination of keywords: “ozonated AND oil [Title/Abstract]” and “ozonized AND oil [Title/Abstract] “. Both terms, ozonated and ozonized, are commonly used as they are both accepted in English written research works. The narrative approach was adopted by synthesizing and critically interpreting the available literature on ozonated vegetable oils. Rather than focusing on quantitative comparison, this approach allowed the identification and comparative discussion of current methodological limitations and potential applications, while also highlighting key research gaps across the available evidence.

### Inclusion and Exclusion Criteria

**Figure 1:**
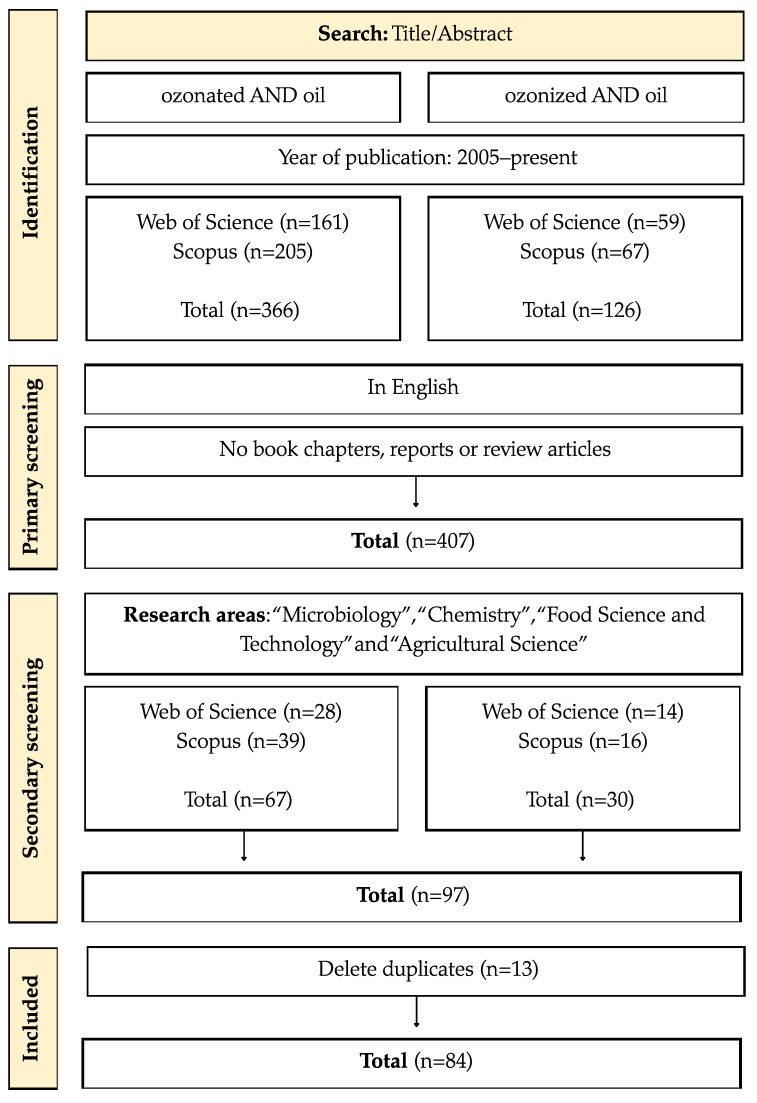
PRISMA flowchart diagram of the followed search strategy and results.

The screening of the papers was carried out based on different criteria reflected in Figure 1. Studies not written in English and not published between 2005 and 2026 were excluded from all databases. Later on, book chapters, reports and review articles were also rejected, including only research work papers. To exclude medical, engineering, pharmaceutical, clinical, veterinary, and other studies not related to the food industry, non-relevant Research Areas were discarded during the screening process. Subsequently, the Topics available in Scopus and Web of Science were grouped into 11 thematic clusters, from which only those corresponding to the Research Areas “Microbiology”, “Chemistry”, “Food Science and Technology”, and “Agricultural Sciences” were selected. The complete clustering strategy and selection criteria are detailed in Supplementary Material SI. Then, all papers were collected and entered into Zotero. After that, duplicate papers were eliminated. The PRISMA flowchart diagram comprehensively illustrated the identification, screening, and selection process of the 84 included articles (Figure 1).

## 3. Results and Discussion

### 3.1. Bibliometric Data

**Figure 2:**
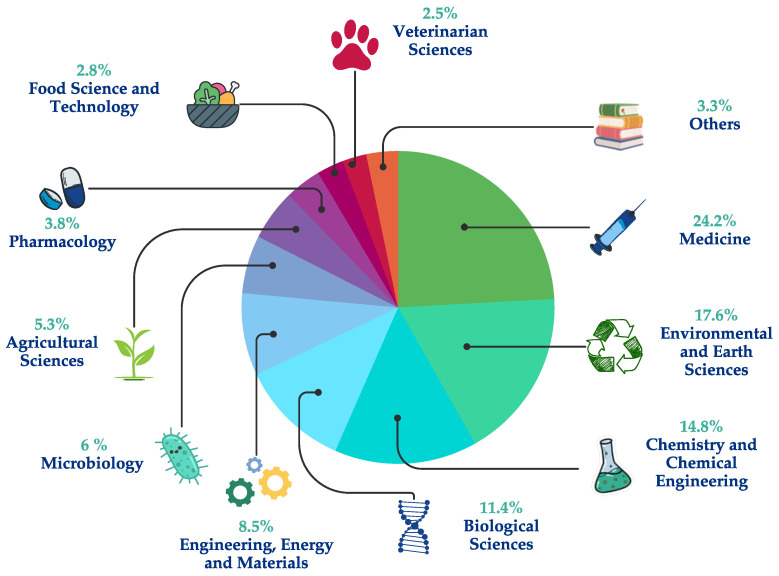
Distribution of research areas/categories of the selected publications according to Scopus and Web of Science and after clustering (Supplementary Material SI), expressed as percentages.

After the primary screening steps (Figure 1), 407 articles fit the search strategy. Although not all these articles were selected for the final review, it was considered interesting to analyze the main research areas in which works about ozonated vegetable oils were published (see Figure 2).

After the initial bibliographic search, approximately one quarter of the studies (24.2%) were related to the medical field, confirming that ozonated oils are mainly investigated for their therapeutic potential, particularly due to their antimicrobial, anti-inflammatory, and wound-healing properties. In fact, there is abundant scientific evidence demonstrating that the topical application of ozonated vegetable oils can be used to treat different dermatological disorders, such as diabetic foot ulcers [24,25,26], wounds [27,28], ulcers [27,29,30], burns [31] and melanoma [32].

In the field of Environmental Sciences (17.6%), the majority of the studies are not focused directly on ozonated oils as therapeutic products, but rather on the application of ozone—mainly in the form of ozonated water—as an advanced oxidation process for the treatment of wastewater. Ozone is widely used to degrade organic pollutants, including oils, fats, surfactants, dyes, pharmaceuticals, and industrial residues, due to its high oxidative potential. In this context, ozonation promotes the breakdown of complex organic molecules into smaller, more biodegradable compounds, thereby improving water quality and facilitating subsequent biological treatment processes. Therefore, the strong representation of Environmental Sciences reflects the relevance of ozone as an effective and environmentally friendly technology for water purification and remediation, rather than a direct interest in ozonated oils themselves [33]. They are also used to improve the thermal and oxidative properties of biodiesel, as ozonated vegetable oils (1%) enhance fuel properties such as pour point, flash point, oxygen content, combustion enthalpy, thermal and oxidative stability, combustion efficiency, and reduce carbon residue [34].

The third major category is Chemistry (14.8%), which reflects the fundamental interest in understanding the chemical reactions involved in the ozonation process, including the formation of ozonides, peroxides, and other oxygenated species, as well as their stability, reactivity, and analytical characterization.

**Figure 3:**
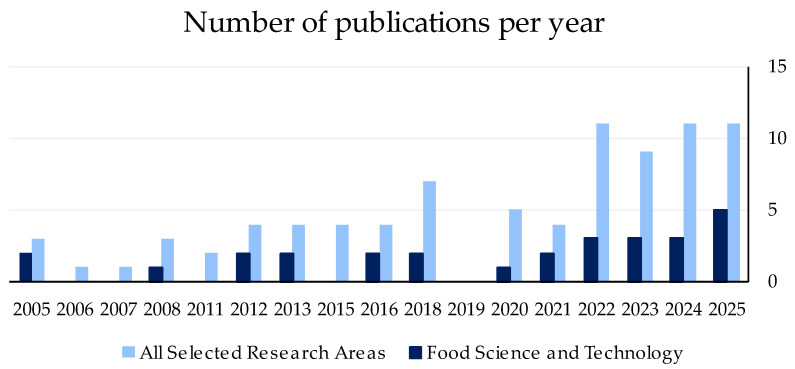
Annual number of total publications (*n* = 84) on ozonized and ozonated oils retrieved from Scopus and Web of Science. Bars in light blue represent the total number of the selected publications per year in all selected research fields (Chemistry, Microbiology, Agricultural Sciences and Food Science and Technology), while the bars in dark blue indicate the number of publications corresponding specifically to the Food Science and Technology category.

Together, the three areas account for more than half of the total scientific production (56.6%), indicating that research on ozonated oils is driven both by applied needs (medicine and environmental remediation) and by basic chemical studies. Although Food Science and Technology alone represents only a small fraction of the literature (2.8%), when combined with related fields such as Chemistry (14.8%), Microbiology (6.0%) and Agricultural Sciences (5.3%), a substantial portion of the available research is covered (see Figure 2). This highlights that, even if food-related applications are not the dominant focus, they are strongly supported by interdisciplinary research addressing chemical mechanisms, microbial control, and agricultural practices, which are essential for understanding the potential of ozonated oils in food preservation and safety. In addition to the distribution by research areas, the temporal analysis of publications reveals a clear increasing trend in the number of studies over time (see Figure 3), particularly within the field of Food Science and Technology. Notably, in the most recent year considered (2025), publications in Food Science and Technology account for approximately half (45.45%) of the total number of selected studies. This reflects a growing interest in food-related applications, in line with the general upward trend observed across all research areas since 2021, as discussed in the previous section.

Among the 84 selected studies, sunflower oil and olive oil were the most frequently investigated oil matrices, with 22 (25.6%) and 21 (24.4%) publications, respectively, together accounting for approximately half of the total literature. A considerable number of studies focused on ozonated oils derived from nuts, including hazelnut, peanut and Brazil nut oils (7 studies, 8.1%), as well as from grains such as soybean, flaxseed and rice (4 studies, 4.7%). In addition, a smaller number of publications addressed oils from fruits of particular interest, such as coconut (1 study, 1.2%), avocado (1 study, 1.2%) and palm oil (3 studies, 3.5%), while the remaining works involved a variety of minor or less commonly used oil sources.

### 3.2. Chemical Nature and Production Process of Ozonated Vegetable Oils

Ozone (O_3_) is a triatomic molecule with great oxidizing and electrophilic properties. This gaseous molecule is able to react with carbon–carbon double bonds (C=C) present in unsaturated fats like oleic, linoleic and linolenic acids, which are abundant in vegetable oils such as olive, sunflower, canola, hazelnut or soybean. The fundamental reaction pathway is described by the classical Criegee mechanism [35], in which ozone attacks carbon–carbon double bonds to form an unstable primary ozonide (1,2,3-trioxolane) that rapidly decomposes into carbonyl fragments and highly reactive carbonyl oxide intermediates. These intermediates may rearrange into secondary ozonides (1,2,4-trioxolanes) under relatively anhydrous conditions or react with water to generate hydroxyhydroperoxides that further decompose into aldehydes and hydrogen peroxide, while parallel pathways yield hydroperoxides, peroxides, polyperoxides, carboxylic acids and other reactive oxygenated species (ROS) capable of initiating lipid peroxidation [36].

These ozonation-induced reactions occur in parallel with autooxidation processes, which proceed via free radical chain mechanisms involving induction, propagation and termination stages, and are accelerated by light, temperature, metal ions and oxygen availability. The coexistence of ozonolysis and autooxidation pathways results in highly heterogeneous chemical systems whose characterization is challenging due to the wide diversity of transient and stable oxidation products [37]. Nevertheless, these reactive oxygen species are directly responsible for the functional properties of ozonized oils, including antimicrobial activity via lipid peroxidation and protein denaturation and potential modulation of inflammatory and oxidative stress pathways in host tissues. The kinetics of these oxidative transformations are highly operational and oil-specific and cannot be described by universal models, requiring individual characterization analyses to predict quality changes and optimize processing and storage conditions [38,39].

The production of ozonated oils typically involves bubbling a controlled stream of ozone-enriched gas through liquid vegetable oil under defined operational conditions. This relatively simple process is, however, governed by multiple interacting parameters, including ozone concentration, gas flow rate, reaction time, temperature, lipid composition and reactor configuration, all of which critically determine the reaction kinetics and the final chemical profile of the product.

A study driven by Dominguez-Lacueva et al. [40] evaluated the effect of ozonation time from 0 to 48 h on oil quality and showed that ozone dosage (range of 0–1 mol O_3_) was the main factor governing oxidation, explaining 81.8% of the variability in both physicochemical properties and antimicrobial response. Similarly, Enjarlis et al. [41] reported a clear dose-dependent increase in oxidation, with a final dosage of 440 mg O_3_ L^−1^, they observed higher microbial reduction together with increased oxidation intensity and viscosity. These results confirm that ozone dose is the primary driver of quality changes during oil ozonation. Reported ozonation conditions in the literature span several orders of magnitude, with ozone production ranging from less than 1 mg O_3_ L^−1^ to more than 70 mg O_3_ L^−1^, flow rates from 0.06 L/min to industrial-scale systems of 30 L/min, and treatment times from a few minutes to several days. This extreme heterogeneity highlights the lack of standardized protocols and severely limits direct comparison among studies.

Temperature also plays a dual role, affecting both ozone solubility and reaction kinetics. Lower temperatures generally favor ozone dissolution in the lipid phase and promote controlled ozonide formation, whereas higher temperatures accelerate decomposition reactions and may enhance the formation of secondary oxidation products. As a result, most experimental protocols (27 out of 84) operate at room temperature (18–25 °C) to preserve ozone stability and ensure reproducible reaction pathways. At lower temperatures, ozone exhibits higher solubility and longer lifetime in the oil phase, favoring controlled formation of ozonides and limiting secondary decomposition reaction. Therefore, when developing delivery systems such as coatings, nanoemulsions, or niosomes, low temperatures (from 0 to 5 °C) are commonly employed, as they play a critical role in maintaining compound stability and solubility, which directly influence encapsulation efficiency and overall system performance, as reported by Fahmy et al., Severino et al., and Shi et al. [32,42,43]. Only a very limited number of studies have explored ozonation at below zero temperatures, with just two articles in the literature reporting processes carried out at −8 °C in peanut oil [44] and −4 °C in mustard oil [45]. Apart from operational temperature, storage temperature also significantly affects the stability of ozonated oils, as reactive oxygen species such as ozonides and hydroperoxides continue to evolve after processing. Low temperatures slow down their decomposition and help preserve antimicrobial activity, whereas storage at room or higher temperatures accelerates secondary oxidation, leading to loss of active compounds and increased formation of aldehydes and acids [46]. Consequently, temperature is a key factor controlling shelf life, although it is often poorly reported in the literature.

The presence of water in the system has been shown to significantly alter ozonation outcomes [47,48]. Water can facilitate ozone decomposition into hydroxyl radicals, thereby introducing additional oxidative pathways that accelerate fatty acid degradation and acid formation. Comparative studies between dry and hydrated systems demonstrate that aqueous environments promote faster oxidation and yield oils with higher acidity and altered chemical profiles.

Although Moureu et al. [48] did not observe significant differences in the antimicrobial activity of ozonated oils obtained from different lipid profiles; they reported marked variations in the degree of oxidation and acidity. Similar observations were made by Balea et al., Díaz et al., and Sadowska et al. [49,50,51] who demonstrated that, even under identical ozonation conditions—temperature, ozone dose and exposure time—, different oils developed substantially different oxidation levels. These differences were not only associated with the type of oil and its fatty acid composition, but also with the degree of refining, as highlighted by Domínguez-Lacueva et al. [40] who showed that pomace and virgin olive oils exhibited distinct oxidative responses to ozonation. Together, these findings indicate that the chemical outcome of ozonation is strongly influenced by both the intrinsic lipid profile and the processing history of the oil, even when biological activity appears comparable.

Finally, reactor design and operational parameters strongly influence the efficiency and outcome of oil ozonation. Gas–liquid contact efficiency, controlled by bubble size, stirring speed, and gas residence time, directly determines ozone mass transfer and reaction kinetics. For instance, Díaz et al. [38] showed that the same amount of double bonds could be consumed under different reaction times depending on the gas source, requiring 5 h when ozone was generated from pure oxygen and 8 h when air was used, highlighting the importance of oxygen purity and gas quality. Similarly, Gu et al. [47] reported that increasing stirring speed significantly improved ozonation efficiency, while optimizing ozone ventilation time reduced energy consumption and overall processing costs. Chemical conditions also play a role, as Enjarlis et al. [41] identified pH 4 as the optimal condition for rice bran oil ozonation, indicating that acidity can modulate ozone reactivity and stability. Together, these examples illustrate that ozonation efficiency depends not only on nominal ozone concentration, but also on gas composition, mixing dynamics and physicochemical environment, which are often insufficiently controlled or reported in the literature.

A major limitation in the current literature is the inconsistent and often incomplete reporting of key parameters such as ozone flow rate, effective ozone dose and reaction temperature. In many studies, one or more of these variables are poorly described or entirely omitted, which severely hampers reproducibility and makes direct comparison between experimental results unreliable. This lack of methodological rigor complicates the interpretation of biological and technological outcomes and highlights the urgent need for standardized reporting protocols and a more transparent description of ozonation procedures in future research. A critical point is that many of the cited studies report ozonation times without providing the applied ozone dose (mg O_3_ per mass or volume of oil), which is essential to meaningfully compare the conversion of C=C bonds to ozonation products. Without this information, comparisons across studies are unreliable. If the authors aim to draw comparisons, the actual ozone doses should be calculated from the original data and explicitly presented. This issue has been highlighted by van Leeuwen [52], who emphasizes the importance of accurately measuring ozone dosage.

### 3.3. Physicochemical and Quality Parameters of Ozonated Vegetable Oils

**Figure 4:**
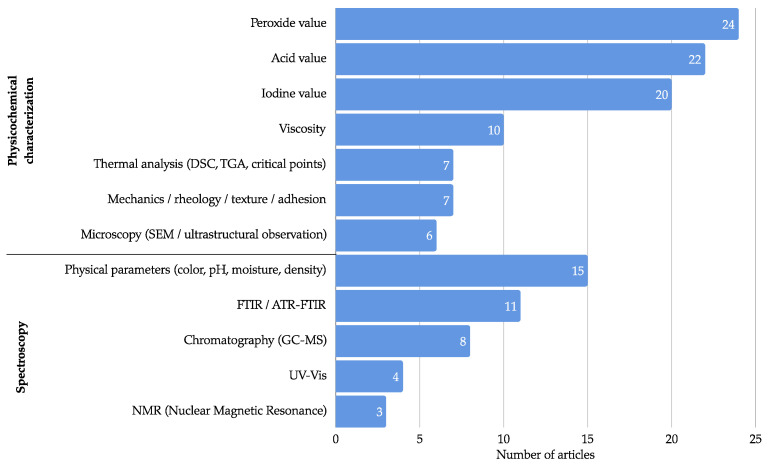
Summary of some of the analytical methods used in the 84 articles selected to characterize ozonated vegetable oils, where DSC method refers to Differential Scanning Calorimetry; TGA is Thermogravimetric Analysis; SEM refers to Scanning Electron Microscope; FTIR to Fourier Transformed Infra-Red spectroscopy; GC-MS method refers to Gas Chromatography-Mass Spectrometry and NMR to Nuclear Magnetic Resonance.

In the context of food conservation, monitoring physicochemical and quality parameters of ozonated vegetable oils is particularly important, as these indicators directly reflect oxidation intensity, stability and antimicrobial potential, which are critical for ensuring both safety and effectiveness. Parameters such as peroxide value, acidity, viscosity and iodine index are widely used to track the progression of ozonation and to avoid excessive oxidation that could compromise sensory quality or generate undesirable by-products. Systematic control of these parameters enables optimization of processing conditions and supports the development of ozonated oils as reliable, safe and reproducible preservation agents for food applications. Spectroscopic and chromatographic techniques such as FTIR, NMR and GC–MS provide detailed structural information on ozonides, peroxides, aldehydes and other oxidation products, allowing direct assessment of reaction pathways and product distribution. These advanced methods are essential for mechanistic understanding and precise chemical characterization, whereas conventional quality indicators mainly offer indirect information on oxidation degree. In addition to chemical and analytical indicators, sensory-related parameters such as color, pH, odor and taste are also relevant quality attributes, particularly for food conservation applications. Figure 4 summarizes the most repeated physicochemical parameters and the advanced techniques used for ozonated olive oil quality assessment in the 84 selected studies.

#### 3.3.1. Physicochemical Quality Analyses

Figure 4 shows that physicochemical parameters are the most frequently assessed indicators in ozonated oil characterization, with peroxide value, acid value, and iodine value being the dominant metrics reported across studies. These parameters appear in 24, 22, and 20 articles, respectively, highlighting their central role in monitoring oxidation progress and chemical transformation during ozonation. Additional factors such as viscosity and general physical attributes (color, pH, moisture, density) are also commonly evaluated, reflecting their importance for determining product stability and functional quality. Overall, the data indicate that physicochemical measurements constitute the primary analytical approach for assessing ozonated oils.

Peroxide value (PV) is the number that expresses, in milliequivalents of active oxygen, the quantity of peroxides contained in 1000 g of oil [53]. This parameter serves as a key indicator for monitoring the ozonation process by quantifying oxidative compounds generated during the ozonation reaction [37]. By tracking changes in peroxide value, researchers can evaluate the effectiveness of ozonation, identify optimal conditions, and ensure that the desired level of modification is achieved, thereby maintaining the product’s quality. Higher ozonation times and concentrations have been demonstrated to be related to higher peroxide values [37]. However, the ozonation conditions together with the olive oil type are two factors that significantly influence the final PV. For example, Sadowska et al. [51] ozonized sunflower oil for 7 h and obtained a 28 mEq O_2_/kg peroxide value, whereas Díaz et al. [54] did it for 2 h, and obtained a 650 mEq O_2_/kg value. The difference between the two lies in 1) the amount of ozone generated and 2) the ozonator used in each study. Additionally, and also for sunflower oil, obtained PVs between 0 and 800 mEq O_2_/kg when including 5% water in the ozonation process; while, when they doubled the water amount to 10%, ozonated sunflower oil achieved PVs between 0 and 1600 mEq O_2_/kg. This suggests that, besides the ozonation equipment and the applied ozone concentration, other factors, such as water, can also influence the reaction between vegetable oils and ozone. In fact, water helps in the diffusion of ozone within the oils and promotes contact with double bonds. Not only water, but also the air source supplying the equipment can affect the reaction performance. However, Díaz et al. [38] showed that using the same sunflower oil and the same O_3_ dosage, the reaction exhibited a perfectly linear relationship regardless of whether air or pure oxygen was used as the source (R^2^ > 99% in both cases).

On the other hand, Balea et al. [49] applied the same ozonation treatment to three different oils (olive, hemp and coconut) and obtained significantly different peroxide values (108.4, 228.4, 68 mEq O_2_/kg, respectively). Similarly, Sadowska et al. [51] demonstrated that, under the same ozonation conditions, soybean oil (37 mEq O_2_/kg) had a higher peroxide index than sunflower oil (28 mEq O_2_/kg). Likewise, Díaz et al. [50] applied O_3_ doses ranging from 0 to 0.97 mg/g in five different oils—dendê (D), soybean (S), corn (C), rice (R), and sunflower (S)—and once again demonstrated that the initial composition of the oil dictates the behavior of its physicochemical properties during ozonation. They observed distinct PVs for each oil (D: 1746, S: 1074, C: 1205, R: 1098, and S: 1350 mEq O_2_/kg), highlighting the influence of the oil’s composition on the ozonation process.

However, the peroxide value becomes unreliable for comparing highly ozonated oils (PV > 1000 mEq O_2_/kg), because it reaches a plateau as the reaction progresses and unsaturated double bonds are progressively depleted, limiting further changes in the system measurable by this assay. At this point, it must be clarified that the AOCS iodometric peroxide value method is primarily based on the assumption that lipid hydroperoxides oxidize iodide (I^−^). However, during ozonation, the reaction does not solely produce classical hydroperoxides, but a complex mixture of oxygenated compounds, including ozonides (1,2,4-trioxolanes), peroxyacetals, polyperoxides, and other secondary intermediates derived from ozonolysis. All of these species contain peroxide-like structural motifs capable of oxidizing iodide under the assay conditions, thereby increasing the amount of iodine formed in the iodometric titration and producing elevated PVs that are not linearly related to classical hydroperoxide accumulation. In the study described by Zanardi et al. [55], peroxide value increased rapidly during the initial ozonation phase (from 2480 mg to approximately 3800 mg O_3_ applied), but subsequently showed a marked reduction in growth rate, even when the ozone dose was further increased up to 9900 mg O_3_. As ozonation proceeds, remaining double bonds become sterically hindered and less reactive, while polymeric peroxides are formed, limiting further measurable peroxide formation. Consequently, ozonation efficiency decreased from 96% at early stages to 31% at advanced stages, demonstrating that peroxide value systematically underestimates the real amount of ozone incorporated and cannot reliably reflect oxidation degree in massively ozonated systems. Consequently, chromatographic methods or spectroscopic techniques such as FTIR and NMR are essential to complement the characterization of ozonated oils, as they allow direct detection of ozonides, peroxides and structural modifications, providing a more accurate and holistic characterization of ozonated systems [56].

In the same way that ozonation causes the formation of oxidized compounds, it also causes the degradation of triglycerides present in the oil, releasing free fatty acids that increase acidity. The acidity value is the number of mg of sodium hydroxide required to neutralize the free acids in 1.0 g of the substance [57]. Similarly to the PV, the acidity value increases as the ozone exposure time and concentration increase [49,51]. The results of the selected studies suggest that there is a correlation between the PV and the AV, given that Balea et al. [49] obtained a higher AV in the ozonated hemp oil (7.84 mg KOH/g oil), whose peroxide value 228.4 mEq O_2_/kg) was also the highest of the three oils (olive, hemp, coconut) used. In the same way, Sadowska et al. [51] obtained a higher AV in soybean oil (1.9 mg KOH/g oil) than in sunflower oil (1.7 mgKOH/g oil), since for sunflower oil the PV was also higher. Despite Ledea-Lozano et al. [37] obtained similar peroxide values in all its ozonated sunflower oils, the common sunflower oil obtained a greater increase in acidity values (from 6.0 to 9.7 mg KOH/g oil) than the palmitic, oleic, or stearic acid-enriched sunflower oils. Díaz et al. [50] also reported marked differences in acidity values among different oils treated under the same ozonation conditions, with AVs ranging from 6.3 mg KOH/g oil in corn oil to 21.0 mg KOH/g oil in dênde oil. These findings confirm that, as with the peroxide value, the acidity value is largely influenced by the initial composition of the oil but is also strongly dependent on the applied ozone dose. Although Díaz et al. [38] initially observed a weaker linear correlation between the ozone dose and the acidity value, this was because the sunflower oils under study were ozonated under different experimental conditions. Oils treated with air required longer reaction times (8 h) than those treated with pure oxygen (5 h), which allowed more time for the decomposition of peroxidic species and further formation of carboxylic acids. This phenomenon becomes more evident over time. In fact, Dominguez-Lacueva et al. [58] demonstrated that, 30 days after ozonation, the peroxide value decreased due to the degradation of formed hydroperoxides and ozonides into carboxylic acids. As a result, the acidity value increases gradually over time and is more pronounced at room temperature (25 °C). In a subsequent study [50], where different oils (dênde, soy, corn, rice and sunflower) were exposed to the same ozonation conditions, a much stronger linear correlation was observed (R^2^ > 91%) between ozone dose and acidity value. This finding was later confirmed by Dominguez-Lacueva et al. [40], who showed, through principal component analysis (PCA), a strong correlation between acidity and peroxide values.

Another change that happens during the ozonation process, as O_3_ concentration and exposure time increase, is a decrease in the iodine value (IV) [49,59]. The IV is defined as the mass of iodine (in grams) that can be absorbed by 100 g of a fat or oil [60]. It quantifies the degree of unsaturation of the fatty acids present in the fat or oil. This reduction occurs because ozone oxidizes the double bonds, leading to fewer unsaturated fats and a lower iodine value [49]. Inversely proportional to PV and AV, the IV is lower the higher the AV and PV are [51]. Díaz et al. [38] reported strong linear correlations between applied ozone dose and the iodine value. In a later study, Díaz et al. [50] found that the reduction in double bonds varied among five oils. Dendê oil showed the most significant decrease (94%) despite its lower initial IV; while the decrease in soy, corn, rice, and sunflower oils ranged from 50% to 60%, suggesting that initial composition influences reactivity. Phuah et al. [61] further confirmed the drastic drop in IV, with nearly a 90% decrease after 24 h of ozonation. Domínguez-Lacueva et al. [40] observed similar trends: in virgin olive oil (VOO), the IV decreased by 44.4, and in pomace olive oil (POO), by 47.5 after 48 h. These findings confirm that, while the extent of IV reduction is influenced by oil type and ozonation duration, it also depends on the availability of double bonds and the specific experimental conditions applied, similarly to other physicochemical parameters. A recent study [58] demonstrated that the main decay in the number of C=C double bonds, and subsequent iodine value decrease, occurs within the first 24 h of ozonation, suggesting that the ozonolysis reaction takes place during the first hours of O_3_ treatment. The IV thus remains a reliable indicator for tracking double bond saturation and evaluating ozonation effectiveness.

Density and viscosity values are frequently studied in ozonated vegetable oils, and they both increase as the ozonation process progresses [49,51,62,63], similar to PV and AV. Whereas viscosity measures the resistance of a fluid to a flow, density quantifies the mass of oil per unit of volume. On one hand, the increase in viscosity is related to a decrease in the flexibility of the ester chains as a result of the consumption of C=C double bonds during the ozonation process [62,63]. On the other hand, the increase in viscosity is related to an increase in the peroxide value of the ozonated oils [49] due to the formation of polyperoxides (described in the Criegee mechanism) with higher molar masses [51,62]. According to Guerra Blasco et al. [62], oil density changes during ozonation relate to added oxygen, with greater density reflecting an increase in peroxide index and oxygen content.

A critical implication of these findings is that, although increasing peroxide value is often associated with enhanced antimicrobial efficacy, excessive oxidation compromises the regulatory and nutritional quality of edible oils. According to Codex Alimentarius Commission (CODEX STAN 33-1981 for olive oils) [64], the peroxide value for extra virgin and virgin olive oils must not exceed 20 mEq O_2_/kg, while free acidity (expressed as oleic acid) is limited to ≤0.8% for extra virgin and ≤2.0% for virgin oils. Additionally, the iodine value for olive oil typically ranges between 75 and 94 g I_2_/100 g, reflecting its characteristic degree of unsaturation. These limits highlight that, despite the technological advantages of ozonation, there is a narrow operational window in which oxidative modifications can be beneficial without exceeding legal thresholds. Therefore, optimization of ozonation conditions is essential to balance antimicrobial effectiveness with compliance to quality standards, ensuring the oil remains suitable for human consumption.

A possible strategy to mitigate the detrimental oxidative and sensory effects of direct oil ozonation is to apply ozone at earlier processing stages, such as on intact raw materials. In this context, the study by Ortega Sanchez et al. [65] on peanut kernels suggests that matrix integrity and process conditions (e.g., relative humidity, ozone concentration, and kernel bed configuration) can modulate ozone reactivity. Their results showed that, despite some changes in color parameters and peroxide value, free fatty acid levels remained stable, maintaining oxidation markers within acceptable limits, indicating a lower degree of lipid degradation compared to direct oil treatment. This supports the idea that ozonating whole kernels may act as a protective approach, limiting excessive oxidation while still enabling microbial control and potential quality preservation.

#### 3.3.2. Chromatographic Techniques

A highly effective and widely used technique for monitoring the ozonation process is the analysis of the lipid profile of vegetable oils before and after treatment using gas chromatography (GC). Fatty acid identification and quantification are performed after a transesterification process with methanol, which produces volatile fatty acid methyl esters (FAMEs) that are easily detectable by GC. This approach enables the characterization of unmodified FAMEs in untreated oils while revealing the formation of new compounds after ozonation.

One of the main effects of ozonation is the progressive depletion of unsaturated fatty acids, particularly oleic and linoleic acids, due to their susceptibility to ozone attack. This reduction depends on both the oil composition and the processing conditions. For instance, Balea et al. [49] reported that untreated olive, hemp, and coconut oils contained 75.71%, 99.96%, and 90.36% fatty acid methyl esters, respectively, whereas ozonation by-products accounted for 38.84%, 26.52%, and 14.41% of the total composition after treatment. Similarly, Ledea-Lozano et al. [56] observed that oleic acid content in high-oleic sunflower oil decreased from approximately 80% to 40% after ozonation, while in sunflower oils richer in saturated fatty acids (palmitic and stearic), oleic acid depletion was even more pronounced, dropping from 70% to 16%. In agreement, Phuah et al. [61] found a strong negative correlation (r = −0.90) between oleic acid concentration and ozonation time. Differences in oil matrices also play a key role: Dominguez-Lacueva et al. [58] showed that virgin olive oil and pomace olive oil, despite similar initial fatty acid compositions, exhibited different reductions in oleic acid (69% and 47%, respectively), suggesting that minor components and extraction-related factors influence ozonation behavior. Likewise, Enjarlis et al. [41] reported that linoleic and linolenic acids were depleted earlier in rice bran oil, while lauric acid (C12:0) was also reduced under acidic conditions (pH 3–4), likely due to structural susceptibility to nucleophilic attack by ozone and interactions with the surrounding medium.

As unsaturated fatty acids are consumed, new oxidation products are progressively formed, and their diversity increases with ozonation time. Al-Rajhi et al. [45] observed an increase in detectable compounds from 10 to 29 after ozonizing mustard oil using GC-MS. Accordingly, Bazaid et al. [66] detected through GC-MS that raw lettuce oil contained 16 molecules spanning 10 chemical classes, whereas the ozonated lettuce oil contained 22 molecules across 12 classes. Similarly, studies on peanut oil [44] showed that both untreated and ozonized samples shared compounds such as 2,4-decadienal (E, E), n-hexadecanoic acid, 9,12-octadecadienoic acid (Z, Z), and oleic acid, although their peak areas decreased after ozonation. According to the Criegee mechanism, the initial reaction between ozone and double bonds leads to the formation of primary ozonides, which decompose into characteristic products such as nonanal, nonanoic acid, azelaic acid, methyl-9-oxononanoate, and monomethyl nonanedioate, typically within the C8–C12 range [56]. These primary products appear at early stages of ozonation, whereas secondary compounds—such as nonenal or caprylic acid—tend to form at higher ozonation levels. For example, Vieira et al. [46] detected compounds including nonanoic acid, azelaic acid, nonenoic acid, dimethyl acetal, and nonanal, while more advanced oxidation led to the appearance of additional species (9-Decen-2-ol, ocatonic acid or octanol acetate). Gu et al. [47] also identified azelaic and caprylic acids, attributing their formation to both direct oxidation of double bonds and free radical-induced cleavage of ester bonds during the ozonation process.

In other studies [56,67], triacylglycerols (TAGs), the primary molecules undergoing chemical changes during ozonization, were monitored using high-performance liquid chromatography (HPLC) with size-exclusion mode, which allows the separation and quantification of TAGs, as well as their transformation products such as dimers, oligomers, and altered TAGs based on their molecular size. Ozonation of sunflower oils led to the immediate formation of TAG dimers following a linear trend proportional to ozone incorporation, indicating a direct (non-radical) mechanism, while oligomers formed progressively and accelerated at later stages due to condensation of smaller species. Altered TAGs reached ~20% in common sunflower oil and ~30–35% in high-oleic variants [56], whereas during storage (1–3 months at 5 °C) polymerized species (dimers and oligomers ~15%) decreased to ~10% due to peroxide bond decomposition, with non-polymerized TAGs increasing to ~80% [67].

Finally, mechanistic studies further support these observations. Zahardis et al. [68] reported that ozonolysis of oleic acid produces characteristic ions (m/z 141, 157, 171, and 187) corresponding to the decomposition of primary ozonides (1,2,3-trioxolanes), confirming the formation of classical Criegee intermediates. Altogether, these findings demonstrate that ozonation not only reduces the content of unsaturated fatty acids but also generates a complex and evolving mixture of oxidation products, whose composition depends on both the reaction conditions and the intrinsic properties of the oil matrix.

However, Ledea-Lozano et al. [37] stated that some of the ozonides and peroxides produced during ozonization split due to the derivatization process of the samples. Moreover, some of the C6 fragments were not detectable because they are highly volatile and, probably, were not properly detected. Thus, it is important to combine these studies with more accurate spectroscopic techniques.

#### 3.3.3. Spectroscopic Techniques

Spectroscopic techniques such as FT-IR, ^1^H-NMR, and ^13^C-NMR offer unique advantages over chromatography by providing critical structural and functional information about molecules, including identification of functional groups, determination of molecular structure, and insights into molecular interactions and dynamics. These techniques have been utilized in 11 out of the 84 studies selected in this category, highlighting their significance in providing essential structural and functional insights.

Before the ozonation process, vegetable oils are characterized by the presence of two broad and strong bands at 1654 cm^−1^ and 3009 cm^−1^, corresponding to C=C and =C-H stretching, in the FT-IR spectra [62,63]. In [37], the most notable spectral changes included the decrease in the =C–H stretching band (~3003–3009 cm^−1^), confirming the consumption of double bonds, and the appearance of a broad band at 3100–3700 cm^−1^, attributed to hydroperoxides and hydroxyl groups formed during ozonation. As the ozonation reaction progresses, the intensity of those two bands decreases, and two new bands appear at ~1099–1109 cm^−1^ and ~1727–1750 cm^−1^. The first band is assigned to the C-O stretch of ozonides [21,62,63] associated with intermediates of the Criegee mechanism [68], whereas the second band represents the broadening of the carbonyl region of C=O, confirming that ozonation leads to double bond cleavage, formation of aldehydes, carboxylic acids and other peroxidic species [66,69]. In addition, a third band (1379 cm^−1^) typical of ozonated vegetable oils appeared in the FT-IR spectra of Georgiev et al. and Guerra Blanco et al. [21,62]. This band corresponds to the trans isomer of the 1,2,4-trioxolane, one of the main ozonides produced during ozonation. Additional bands around 1710 cm^−1^ have been detected by Phuah et al. [61] during the ozonation of more peculiar oils, such as krill and neem oil. This pattern is fairly common as, even in ozonated lettuce oil, the FTIR spectrum [66] showed similar characteristic regions to the native oil, with only minor differences such as the disappearance of a few bands after ozonation (the bands at 2731.26, 1533.51, and 477.83 cm^−1^). The authors suggest that this band corresponds to the formation of functional groups such as aliphatic aldehydes or ketones.

However, even though FT-IR technology permits seeing the transformation of the double bonds into the main ozonides, Georgiev et al. [21] considered that ^1^H-NMR or ^13^C-NMR are the appropriate techniques to identify and quantify both cis and trans isomers of the main ozonation products. It is known that 1,2,4-trioxolane exists in both forms (cis and trans isomers), to a greater or smaller extent, depending on the ozonolysis reaction and the double bond stereochemistry [21]. In ^1^H- and ^13^C-NMR spectra, the signal (δ = 5.3–5.35 ppm 128–130 ppm, respectively) corresponds to the double bonds of oleic, linoleic and linolenic acids [36]. There is a correspondence, just as in FT-IR, between the disappearance of these signals and the appearance of signals corresponding to the formation of ozonides. In the case of ^1^H-NMR, several new signals appear during ozonation: 1.64 ppm for the methylene attached to the ozonide, at 2.04–2.18 ppm for the methylene bonded to the ozonide, and a multiplet signal system at 5.12–5.19 ppm, 5.22–5.33 ppm, and 5.42–5.47 ppm assigned to the ozonide protons [62,63]. For both spectroscopic techniques, it is well-defined that the new signal at 5.1 ppm (^1^H-NMR) [21,37,51,64,65] and 104.5 ppm (^13^C-NMR) [36] corresponds to the formation of the 1,2,4-trioxolane ring proton. This signal (5.1 ppm) has been used as an indicator for the total ozonide content [62], referring to the presence of Criegee ozonolysis products.

However, olefinic protons from hydroperoxides, another byproduct of ozonolysis, could also be detected at δ= 5.55 ppm [37]. Georgiev et al. [21] demonstrated the proportion between cis- and trans- isomers of 1,2,4-trioxolane was of 46:54, in accordance with previous studies. Similarly, Díaz et al. [54] showed that during ozonation the olefinic proton signal (~5.3 ppm) decreases, while new signals corresponding to ozonides (~5.1 ppm) and aldehydes (~9.7 ppm) appear, confirming the formation of oxygenated compounds; whereas ^13^C NMR revealed signals for ozonides (104–122 ppm) and aldehydes (199–203 ppm), supporting the transformation of double bonds into Criegee-type products.

Even though all spectroscopic techniques showed a correspondence between the disappearance of the double bond signals and the appearance of ozonide signals, the consumption of these double bonds is faster or slower depending on the initial composition of the oil. For example, Guerra Blanco et al. [62] demonstrated that grapeseed oil reached the maximum concentration of ozonated compounds after 3 h of ozone treatment. Aligned with these results, Sadowska et al. [51] defined that, under its ozonation conditions, 2.26 h are needed to completely consume the double bonds of oleic acid; whereas, to consume those of linoleic and linolenic acid, 2.65 and 2.95 h were needed.

Overall, these results highlight that ozonated oils constitute a highly complex and dynamic system, composed of a wide variety of molecules with different chemical natures. This composition is strongly influenced by factors such as the type of oil, its fatty acid profile, and the specific ozonation conditions, meaning that no two systems are exactly identical. Consequently, it remains challenging to attribute the observed bioactivity to a single compound or a well-defined group of molecules. Nevertheless, consistent patterns—such as the formation of ozonides (with 1,2,4-trioxolane being the most cited and relevant intermediate), peroxides and oxygenated derivatives—emerge across studies, providing a valuable framework that should be used as a reference for understanding and guiding future research in this field.

#### 3.3.4. Sensory Attributes

Ozonation induces significant chemical and sensory modifications in vegetable oils, with both beneficial and detrimental effects depending largely on treatment intensity and duration. According to Obadi et al. [70], ozone promotes lipid oxidation through reactions with unsaturated fatty acids, leading to the formation of numerous volatile compounds—particularly aldehydes such as hexanal, (E)-2-heptenal, and (E, E)-2,4-decadienal—which are well-known contributors to rancid odors. Their results showed a decrease in linoleic acid content alongside an increase in secondary oxidation products, confirming oxidative degradation. Similarly, Majcher et al. [71] reported that 2 h of ozonation treatment generated key odor-active compounds such as nonanal, (E, Z)-2,6-nonadienal, (Z)-3-hexenal, and hexanal, significantly altering the sensory profile of cold-pressed flaxseed (CPF) oil toward green, cucumber-like, and rancid notes, particularly under prolonged treatment or storage conditions. These findings highlight a major drawback of ozonation: the deterioration of sensory quality due to oxidation.

However, several studies also demonstrate potential advantages of controlled ozonation. Obadi et al. [70] observed a significant increase in total phenolic content (from 6.09 to up to 19.77 mg GAE/g) and antioxidant activity after treatment, suggesting that ozone may disrupt cellular structures and release bound phenolic compounds. This is consistent with findings by Dominguez-Lacueva et al. [72], who reported that despite oxidative conditions, polyphenols and tocopherols remained detectable even after extended ozonation (up to 20 h), indicating a certain resilience of these bioactive compounds.

Likewise, Kaur et al. [73] found increased phenolic content in peanut oil after ozonation and roasting of peanuts, reinforcing the idea that moderate ozonation can enhance antioxidant availability. This effect has already been recorded by Brodowska et al. [74] when using ozone gas during post-harvest. Their study revealed that during short ozone contact times, higher amounts of TPC, 15.47 and 12.91 mg CE/g of extract appeared, whereas high ozonated exhibited the lowest amount of phenolics, 8.77 and 5.18 mg CE/g of extract. Ozone might have the capacity to attack covalent bonds and liberate antioxidants such as carotene, tannin, ascorbate, flavoprotein, and polyphenols from repeating polymers [70].

In contrast, the impact on tocopherols appears more complex. Demirci et al. [75] reported that, while ozonation can increase γ-tocopherol levels—likely due to release from disrupted cellular matrices—, it simultaneously reduces α-tocopherol, especially at higher ozone doses or longer exposure times, since ozone is capable of cleaving double bonds in carotenoid pigments, such as β-carotene, xanthophyll, and flavones [70]. Thus, antioxidant composition may shift rather than uniformly improve.

Regarding the associated effect in sensory properties, particularly color, ozonation generally leads to pigment degradation. Uzun et al., and de Oliveira et al. [36,76] showed that increased ozone exposure causes oils to become paler due to oxidation of carotenoids, with significant reductions in yellow color intensity (b* values). Accordingly, Dominguez-Lacueva et al. [72] showed that the most significant difference between the EEMs of non-ozonated and ozonated virgin olive oil (VOO) was observed in the intense emission band at ʎex/ʎem of 400/675 nm corresponding to the fluorescence of chlorophyll pigments, mainly pheophytins. The absence of pheophytin emission in the EEM of ozonated VOO explains the color change from green/yellowish to white in ozonated vegetable oils. Nonetheless, Bechlin et al. [77] indicated that these changes may be immediate, but not necessarily progressive during storage, suggesting a short-term effect of ozone on visual attributes.

Overall, the literature indicates that ozonation is a double-edged process: while it can enhance antioxidant content and release valuable minor compounds under controlled conditions, excessive treatment promotes lipid oxidation, degradation of key nutrients such as α-tocopherol, and deterioration of sensory and visual quality. It should be noted that the application of ozonated oils in real food systems remains limited, and therefore systematic studies addressing targeted mitigation strategies for odor masking or sensory optimization are scarce in the current literature. Therefore, sensory acceptability should be considered a critical research gap that will ultimately determine the feasibility of their incorporation into food applications.

### 3.4. Antimicrobial Activity of Ozonated Vegetable Oils

The use of ozonated vegetable oils as antimicrobial agents in food systems has gained increasing attention due to their strong oxidative potential and broad-spectrum activity. A wide range of studies (see Table 1) consistently demonstrate that antimicrobial efficacy increases with the degree of ozonation, typically reflected by higher peroxide values (PV).

The application of ozonated vegetable oils as antimicrobial agents in food systems represents a promising but still evolving strategy, particularly when considering the balance between microbial safety and physicochemical quality. A substantial body of evidence demonstrates that antimicrobial efficacy is strongly linked to the degree of oxidation, typically reflected by increasing peroxide values (PV). Overall, Gram-positive bacteria exhibited higher susceptibility to ozonized vegetable oils, with MIC values ranging from ~1.5–8.5 mg/mL and, in highly active formulations, reaching 15.6–62.5 µg/mL, together with larger inhibition zones (≈10–22 mm or higher), whereas Gram-negative bacteria showed higher resistance with MIC values of ~4.5–19 mg/mL and occasional lack of detectable activity. Quantitatively, Gram-negative organisms required, on average, ~5.5 times higher oil concentration (≈5.5-fold increase in MIC) to achieve the same inhibitory effect observed in Gram-positive bacteria, corresponding to ~40–60% lower MIC values and ~30–50% larger inhibition zones in Gram-positive strains, particularly *P. aeruginosa* and *E. coli*, likely due to outer membrane diffusion barriers limiting lipid-derived reactive species.

Studies such as those by Díaz et al. [38,78] reported effective inhibition of pathogens, including *S. aureus*, *E. coli*, and *P. aeruginosa* at PV ranges between ~361 and 2500 mEq O_2_/kg. More recent studies reinforce this trend while expanding its applicability. Díaz et al. [50] demonstrated that oils with higher peroxide values (up to 1746 mEq O_2_/kg) exhibited enhanced antimicrobial activity, although strongly dependent on oil composition.

Similarly, Silva et al., Dos Santos et al., and Soto Beltran et al. [24,79,80] confirmed antimicrobial activity across different oil matrices and bacterial strains, with minimum inhibitory concentrations (MICs) generally decreasing as oxidation increased. Soto Beltran et al. [80] showed that both ozonated olive and venadillo oils were proven to be effective against *E. coli* (ATCC 700609) and *S. aureus* (ATCC 29213).

The main conclusions suggested that there were no significant differences between the two types of oil, whose peroxide values ranged between 643 and 892 mEq of oxygen/kg oil. In addition, they observed greater effectiveness against *S. aureus* (MIC = 1.5 mg/mL) than *E. coli* (MIC = 4.5 mg/mL), concluding that these oils had greater activity against *Gram +* than *Gram −* bacteria.

In the same line, Dos Santos et al. [79] demonstrated that ozonated sunflower oil was effective against *S. aureus* but not against *E. coli*, when tested at concentrations ranging from 1024 to 8 μg/mL—levels chosen to reflect safe in vivo doses for the treatment of infectious diseases. This aligns with the findings of Silva et al. [24], in which ozonated oils—particularly at a concentration of 4.24 mg/g—showed strong antimicrobial activity against both MRSA (methicillin-resistant *S. aureus*) and MSSA (methicillin-susceptible *S. aureus*) strains, with inhibition zones of 15–17 mm.

The consistent susceptibility of different *S. aureus* strains to ozonated oils reinforces their potential as effective agents against drug-resistant pathogens. While conventional antimicrobial resistance arises through mechanisms such as drug inactivation (e.g., β-lactamases), modification of molecular targets, or increased efflux pump activity, ozonated oils act through a fundamentally different, non-specific oxidative mechanism. Ozonides damage microorganisms by disrupting cell walls and cytoplasmic membranes, facilitating their penetration into the cell. Once inside, they oxidize DNA, proteins, and enzymes, impairing essential cellular functions and, ultimately, leading to cell death. As discussed by Al-Rajhi et al. [45], ozonized oils can also inhibit key microbial enzymes and reduce nucleic acid content, further limiting microbial growth.

Likewise, Dominguez-Lacueva et al. [40] identified threshold PVs (~372–575 mEq O_2_/kg) above which antimicrobial effects became significant, particularly against Gram-positive bacteria. This trend has been largely explained by the greater permeability of the peptidoglycan-rich Gram-positive cell wall compared with the lipopolysaccharide outer membrane of Gram-negative bacteria, which limits the penetration and thus the efficacy of hydrophobic ozonated oils.

Since the mechanism is totally not specific, Puxeddu et al. [81] explained their wide-spectrum antimicrobial activity against several microorganisms like: *C. albicans*, *Enterococcus faecalis*, *S. aureus*, *Klebsiella pneumoniae*, *P. aeruginosa*, and *E. coli* or Vieira et al. [46], who added to this list the pathogens *Salmonella choleraesuis, Aspergillus brasiliensis*, and *Malassezia furfur*.

More recent investigations further support the broad-spectrum activity of ozonated oils. Sehim et al. [82] demonstrated strong antifungal activity against *Alternaria alternata*, while Donato et al. [83] reported effectiveness against a wide range of clinically relevant bacteria and yeasts, including *C. albicans* and *K. pneumoniae*, particularly at extremely high PVs (>3000–4000 mEq O_2_/kg). Likewise, Braga et al. [84] confirmed activity against *Pythium insidiosum*, while Al-Rajhi et al. [45] reported remarkably low MIC values (15.62–62.50 µg/mL), suggesting that certain oils may achieve high efficacy even at lower doses.

From an application perspective, some studies have begun exploring the use of ozonated oils directly in food matrices. For example, Ebrahimi et al. [85] demonstrated beef burger shelf-life extension through microbial reduction, suggesting potential as a preservation strategy. Authors performed sensorial, physicochemical and antimicrobial activity studies to analyze the usefulness of these oils in A) Control beef burger, B) Extra Virgin Olive Oil (EVOO) formulated beef hamburger and C) Ozonated EVOO (O-EVOO) formulated beef hamburger. The ozonation procedure consisted of a 5 min ozone treatment of EVOO with a resultant 10.36 ± 0.46 mEq O_2_/kg oil peroxide index. Their findings suggested that O-EVOO was able to extend the shelf-life of the beef hamburger from 3 (normal shelf life at 4 °C) to 15 days of storage due to the significant reduction in TVC (Total Viable Counts), LAB (Lactic Acid Bacteria), *E. coli* and *L. monocytogenes*.

Additionally, the study found that the addition of O-EVOO did not adversely affect sensory properties such as color. This study highlights the potential of O-EVOO as an innovative, natural additive for the food industry, offering enhanced preservation, extended shelf life, and improved safety in meat products and beyond.

Similarly, Guadalupe Armas et al. [86] showed microbial reductions in milk systems, indicating applicability in complex food matrices. They evaluated the efficacy of Ozonated Sunflower Oil (OSO) at three different peroxide index concentrations (150, 300 and 600 mEq O_2_/kg oil) against *E. coli* in goat, cow and sheep milk. The results showed that OSO exhibited effective antimicrobial activity for up to 72 h after application, with a more pronounced effect in formulations with higher peroxide indexes (300–600 mEq O_2_/kg oil). Additionally, the bactericidal efficacy varied depending on the organic matter content of the milk, being most effective in cow’s milk, followed by goat’s milk, and being least effective in sheep’s milk. This study is particularly significant as it presents ozonated vegetable oils as a potential alternative to antibiotics for enhancing food safety and combating infections like mastitis.

In addition, the use of ozonated oils has proven to be effective not only as a fungicide against *Alternaria alternata* on fresh oranges, but also in inhibiting the germination and toxin production of *A. alternata* (ATs) [82]. This finding is significant as it reinforces the previous study, showing that ozonated oils (OZO) can be a sustainable and effective alternative against various pathogens, not just before harvest but also during the storage of fresh produce. The oil used in this study had an exceptionally high peroxide index (1280 ± 2.57 mEq O_2_/kg oil), with a MIC of 0.186 mg/mL and an MFC of 1.57 mg/mL. Furthermore, OZO at a concentration of 5 mg/mL reduced conidia germination by 98% and inhibited the production of three toxins (AOH, AME, and TeA) by 73.4%, 76%, and 67.1%, respectively, at a concentration of 20 mg/mL. These results suggest that OZO could be used as an alternative to traditional fungicides for spraying on fresh fruits.

Nonetheless, contradictory findings exist: Dos Santos et al., and Perpétuo et al. [79,87] reported limited or no antimicrobial effect under certain conditions, highlighting that efficacy is not universal and depends on factors such as microorganism type, oil composition, and oxidation degree.

There are already some commercial products, such as LIQUENSO^®^ Oxygenat (a new agrochemical treatment based on ozonated oleic acid), that are being used as fungicides in wine cultivars. Stahl et al. [88] evaluated not only the high effectiveness of these novel products but also their potential adverse effects on the microbiota of wine grapes. Certain microbial populations, such as acetic acid bacteria (AAB), lactic acid bacteria (LAB), and yeasts from the *Saccharomyces* and non-*Saccharomyces* genera, play a crucial role in wine development. The study found that using this new agrochemical at very low concentrations (0.25% *v*/*v*) had a stronger impact on key strains like *Brettanomyces bruxellensis*, *Saccharomyces cerevisiae*, *Pediococcus* sp., and *Acetobacter aceti* compared to more conventional agrochemicals. Therefore, the authors emphasize the need for careful evaluation of ozonide concentration and toxicity, particularly to assess their long-term effects.

A critical limitation in current research is the lack of standardization. Studies vary widely in ozone dosage, exposure time, oil type, and analytical methods, making cross-comparison difficult. Furthermore, most works focus on in vitro antimicrobial assays, with limited translation to real food systems where factors such as lipid oxidation, sensory deterioration, and consumer acceptance become important. There is also a notable gap regarding toxicological assessment and the stability of oxidation products during digestion.

To sum up, ozonated oils represent a compelling antimicrobial tool with clear potential for food preservation, but their practical implementation needs further research in a wider number of food matrices.

### 3.5. Active Packaging and Antimicrobial Films

Recent advancements in the incorporation of ozonated vegetable oils into biocomposite matrices have demonstrated significant potential for applications in the food industry, particularly as antimicrobial biofilms.

In a recent study [89], Zakrewski et al. evaluated the antimicrobial efficacy of various nanohydroxyapatite (nHAp) formulations. They found that nHAp loaded with Cu^2+^ ions and ozonated olive oil exhibited the highest efficacy, achieving nearly 100% reduction in *Streptococcus mutans* within 4 h. Moreover, the ozonated oil contributed to changes in the surface characteristics and morphology of the nHAp, likely improving its hydrophobicity and stability. This suggests a synergistic effect between copper ions and ozonated olive oil in enhancing antimicrobial properties.

The study of Fahmy et al. [32] investigated the use of niosomal nanovesicles to encapsulate ozonated olive oil, aiming to improve its bioavailability and therapeutic efficacy. This encapsulated formulation exhibited significantly enhanced water solubility, better skin permeation, and a marked increase in anticancer activity—showing twice the efficacy against human melanoma cells compared to free ozonated olive oil. While primarily focused on medical applications, the findings suggest that niosomal encapsulation could be applied to food packaging, where enhanced solubility and controlled release properties could improve the stability and bioavailability of ozonated oils, enhancing their antimicrobial properties in biocomposite films for food preservation.

In another study [90], Khachatryan et al. developed a hyaluronic acid-based hydrogel containing micro/nanocapsules of ozonated olive oil. This biocomposite exhibited regenerative properties and a weak antimicrobial effect against both commensal skin microbiota and pathogenic *Candida*-like yeasts, making it promising for biomedical applications. The study also confirmed that the emulsions had rheological stability and were non-cytotoxic, suggesting safe usage in therapeutic contexts, such as wound healing or skin care products.

However, these studies have paved the way for future research directly applied to the food industry. Nowak et al. [91] demonstrated that chitosan-based films with nano/microcapsules of ozonated olive oil had promising potential for food spoilage prevention. The films exhibited significant antimicrobial activity, with inhibition zones of 26.7 mm against *S. aureus* and 14 mm against *E. coli*. They also maintained good mechanical strength and flexibility, making them suitable for food packaging. Rheological tests revealed that the films had stable shear-thinning properties and could form protective coatings, suggesting their ability to extend the shelf life of food products by inhibiting microbial growth. Moreover, another study [92] also focused on the development of chitosan-alginate films incorporating ozonated olive oil. These films demonstrated pH-sensitivity, changing their optical properties when exposed to acidic or basic environments, which could be used in smart packaging. This packaging could indicate food spoilage or degradation by altering color, offering a practical application in the food industry.

Emulsions are valuable in food packaging because they enhance barrier properties, enable controlled release of active compounds, improve mechanical strength, and offer sustainable, customizable solutions for food preservation. Uzun Karka et al. [93] studied the emulsifying capacity and rheological properties of emulsions made with ozonated hazelnut oil and whey protein isolate (WPI). Their results showed that an ozone exposure longer than 30 min (equivalent to 0.012 g O_3_/mL) negatively affected both emulsifying activity and stability. However, creaming, a common indicator of emulsion instability characterized by the upward migration of dispersed droplets, was found to be slower in emulsions with moderate ozone treatments. The control treatment, which included non-ozonated hazelnut oil, obtained a creaming of 23% after 75 days of storage, while the formulations with hazelnut oil ozonated for 5, 30 and 60 min obtained a creaming of 20%, 18% and 16%, respectively. On the other hand, the accumulation of free saturated fatty acids and products derived from lipid oxidation caused significant changes in the melting curves and crystallization profiles of the emulsions, again, obtaining a negative correlation (*p* < 0.05) between ozonation time and these properties. Therefore, despite the possibility of incorporating emulsions in food coatings or packaging, it is important to conduct further research on the effect of ozonation on such formulations, as both the dosage and application time proved to be highly limiting factors.

Recent advancements in the incorporation of ozonated vegetable oils into biofilms show great promise for food packaging applications, particularly in the development of smart and sustainable coatings that actively help extend food shelf life. However, this technology is still in an early stage of development, requiring further research to validate its stability, scalability, and real-world performance across diverse food systems.

### 3.6. Functional and Toxicological Aspects of Ozonated Oils and Their Implications for Food-Grade Applications

Beyond their antimicrobial capacity, ozonated oils exhibit a wide range of biological activities—including antioxidant and anti-inflammatory effects—that may improve their suitability for food applications. Understanding their toxicological profile and overall safety is therefore essential for defining acceptable exposure levels and ensuring regulatory compliance in food systems. In parallel, the therapeutic and functional properties reported in biomedical contexts offer valuable insights into their biological interactions and potential mechanisms of action. The following table (see Table 2) summarizes the available studies addressing these aspects, providing a comprehensive overview of the evidence relevant to their prospective use as food-grade preservatives.

A growing body of evidence suggests that ozonated oils may offer not only antimicrobial properties but also relevant biological benefits that could support their application in food systems; however, these advantages must be critically interpreted in light of their limitations and translational challenges. Early work by Zamora Rodríguez et al. [94] demonstrated strong gastroprotective effects of ozonated sunflower oil (OSO), with ulcer inhibition reaching 97.4% and restoration of antioxidant enzymes such as SOD and GSH-Px. These findings are particularly relevant because they suggest that, despite being highly oxidized systems, ozonated oils may paradoxically exert antioxidant effects in vivo, likely through the modulation of endogenous defense systems rather than direct radical scavenging.

This dual behavior has been consistently reported in subsequent studies. For instance, Cho et al. [95] showed that high concentrations of OSO maintained macrophage viability (69.1%) and even promoted cell proliferation by reducing ROS and apoptosis, while Radzimierska-Kaźmierczak et al. [18] observed only slight cytotoxicity at high doses in normal cell lines, with negligible effects below 625 μg/mL. Similarly, Kim et al. [96] reported no cytotoxicity and clear anti-inflammatory effects via downregulation of iNOS, COX-2, and pro-inflammatory cytokines. These results collectively support a relatively safe toxicological profile under controlled conditions, which is a key prerequisite for any food-related application.

More importantly, several studies highlight the anti-inflammatory and hepatoprotective potential of ozonated oils. It has been demonstrated [97] that OSO significantly reduced liver damage markers (AST −26.6%, ALT −63.4%), inflammation (IL-6), and lipid accumulation (−46.9%) in zebrafish exposed to glycation-induced stress. Complementarily, Kim et al. [96] linked these effects to modulation of MAPK signaling pathways, suggesting a mechanistic basis for their bioactivity. These findings are particularly relevant when considering them as potential functional ingredients in food systems, as they indicate that ozonated oils may not only inhibit microbial growth but also mitigate oxidative and inflammatory responses associated with foodborne toxins or degraded lipids.

Long-term studies further reinforce their potential safety and functionality. Two recent studies [98,99] demonstrated sustained protective effects over time (2 years), including improved survivability, reduced ROS production, and prevention of age-related degeneration in zebrafish over a two-year period. These results suggest that ozonated oils could be considered not only as preservatives, but also as functional ingredients with added health value. However, it must be emphasized that these studies are largely based on aquatic models, which limits direct extrapolation to human dietary exposure.

Translation to mammalian systems provides additional, albeit more variable, evidence. Cho et al. [100] reported no toxicity after four weeks of OSO supplementation, along with reductions in LDL-C (−36.5%) and liver injury markers. Similarly, Kato et al. [101] observed reduced liver fat accumulation, inflammation (CRP, PAI-1), and lipogenic gene expression in obese rats while Kato et al. [102] reported more moderate or inconsistent effects in mice models. These discrepancies highlight an important limitation: the biological effects of ozonated oils appear to be species-dependent and influenced by metabolic context.

From a food preservation perspective, ozonated oils present a paradoxical yet promising profile: despite being highly oxidized systems, multiple studies indicate that they are non-toxic and even beneficial, as evidenced by improvements in key biomarkers such as reduced inflammation, lower oxidative stress, and enhanced antioxidant enzyme activity. These properties support their potential use as multifunctional agents capable of simultaneously controlling microbial growth and showing potential health-related benefits. However, their strong antimicrobial activity also raises an important and largely unexplored concern regarding their possible impact on the gut microbiota, as non-selective antimicrobial effects could disrupt beneficial microbial populations upon ingestion. In addition, current evidence is largely derived from in vitro and in vivo biomedical models rather than from real food systems. Therefore, although ozonated oils show clear potential as safe and functional ingredients, their practical application in foods requires further research addressing their behavior in complex matrices, long-term toxicological effects, and especially their interaction with the human gut microbiome.

### 3.7. Regulatory Framework of Ozonated Vegetable Oils

The regulatory status of ozonated vegetable oils remains controversial and is directly constrained by their physicochemical properties. Particularly, their elevated peroxide values (300–4000 mEq O_2_/kg), which substantially exceed the established limits for edible olive oils (20 mEq O_2_/kg) by the Codex Alimentarius (CODEX STAN 33-1981). This discrepancy effectively excludes their use as conventional food ingredients across major regulatory frameworks. Notably, studies performed in real food systems generally use lower ozonation levels or reduced peroxide values compared with in vitro studies, reflecting an attempt to balance antimicrobial functionality with product stability and technological feasibility.

Within the European Union (EU), ozonated oils are not recognized as authorized food ingredients under the framework of Regulation (EC) No 1935/2004 and neither as an authorized food additive under Regulation (EC) No 1333/2008. However, Regulation (EU) No 528/2012 approved the use of oxygen-generated ozone as an active substance for biocidal purposes. Thus, their most plausible regulatory pathway would be as processing aids, post-harvest antimicrobial treatments or surface disinfectants, provided that residues are not present in the final food product.

In the United States, the Food and Drug Administration (FDA) recognizes ozone as Generally Recognized as Safe (GRAS) for certain antimicrobial applications in gaseous form; however, this status does not extend to ozonated lipid matrices. Consequently, ozonated oils may be more realistically positioned again as processing aids, post-harvest antimicrobial treatments, or as surface disinfectants.

In China, under the National Health Commission regulatory framework and GB standards governing food additives (e.g., GB 2760-2024) [103], ozonated vegetable oils are not listed as permitted food additives. Their use would therefore be restricted to processing aid applications, particularly in sanitation or surface decontamination contexts.

Overall, across all three regulatory regions, ozonated vegetable oils cannot be classified as edible oil ingredients due to safety and compositional constraints. The most consistent regulatory interpretation positions them either as processing aids or surface decontaminants.

However, research in this field is still at a very early and exploratory stage, and the growing body of data from biomedical applications of ozonated vegetable oils suggests that future regulatory recognition cannot be entirely ruled out—particularly as active components in packaging and, potentially, even as specialized food ingredients under strictly controlled conditions—as scientific knowledge and safety data advance.

## 4. Conclusions

Ozonated vegetable oils represent a compelling and innovative strategy for food preservation due to their strong antimicrobial activity, oxidative stability, functionality, and versatility for incorporation into food systems or active packaging materials. However, their current development is constrained by major scientific and technological challenges. Foremost among these is the lack of standardized ozonation protocols—spanning ozone dosage, treatment duration, temperature, and analytical reporting—, which limits reproducibility and complicates comparison across studies.

Although substantial evidence supports their effectiveness against a broad range of microorganisms and their health-related benefits, most research has been conducted in vitro, and only a few studies have evaluated performance within real food matrices, where lipid oxidation, sensory acceptability, and interactions with food components play critical roles. Safety and toxicological data suggest a generally favorable profile, yet significant gaps remain regarding long-term exposure, digestive stability of oxidation products, and potential effects on gut microbiota.

From a regulatory standpoint, the oxidation levels required for antimicrobial efficacy exceed permissible limits for edible oils, indicating that ozonated oils will need to be regulated as processing aids or components of active packaging rather than as conventional food ingredients. To advance their practical implementation, future research should be focused on developing standardized production frameworks, optimizing ozonation conditions to achieve efficacy at lower oxidation levels, exploring encapsulation or controlled release systems to mitigate sensory impacts, and conducting in vivo and food matrix-based studies that more accurately reflect real-world applications. With these advancements, ozonated oils could evolve into a safe, functional, and sustainable tool for modern food preservation.

## Figures and Tables

**Table 1:** Summary of the antimicrobial activity of different ozonated vegetable oils reported by various authors (where PV means Peroxide Value; ATCC means American Type Culture Collection; STCC means Spanish Type Culture Collection; MRSA means Methicillin Resistant *S. aureus* and MSSA means Methicillin Sensible *S. aureus*; MIC means Minimal Inhibitory Concentration; CFU means Colony Forming Units and Ø means Inhibition diameter).

References	Type of Oil	Ozonation Information	Microorganism	Antimicrobial Activity
[24]	Mixture of extra virgin olive oil and refined sunflower oil	PV = 3.9–220.7 mEq O_2_/kg	28 MRSA and 14 MSSA strains of *S. aureus*	1.06–8.48 mg/g (MIC)
[38]	Sunflower oil (SO)	PV = 361–675 mEq O_2_/kg	*S. aureus* (ATCC 25923), *E. coli* (ATCC 25922) and *P. aeruginosa* (ATCC 27853)	*S. aureus*: 4.75 mg/mL (MIC) *E. coli*: 9.5 mg/mL (MIC) *P. aeruginosa*: 19 mg/mL (MIC)
[40]	Virgin and pomace olive oils	13.2–1255.2 mEq O_2_/kg	*E. coli* (STCC 45)* P. aeruginosa* (STCC 109)* S. aureus* (STCC 239)	Antimicrobial activity started to be noticeable for the three strains at 372.05 and 574.70 mEq O_2_/kg for ozonated virgin and pomace oils, respectively.
[45]	Mustard oil	8.0 g h−1 for 48 h	*S.aureus* (ATCC43300), *E. faecalis* (ATCC51299), *K. pneumonia ATCC70063*, *Salmonella typhi* (ATCC13076) and *fungi Aspergillus niger *(ATCC10864), and *C. albicans* (ATCC90028)	The examined bacteria were suppressed with excellent MIC (15.62 to 62.50 μg/mL) and MBC (15.62 to 125 μg/mL)
[46]	Sunflower oil	PV = 125 meq O_2_/kg	*S. aureus* (ATCC 6538), *E. coli* (ATCC 8739), *Salmonela choleraesuis* (ATCC 10708), *P. aeruginosa* (ATCC 9027), *C. albicans* (ATCC 10231), *Aspergillus brasiliensis* (ATCC 16404), and *Malassezia furfur* (ATCC 14521)	Broad-spectrum antimicrobial activity against all tested strains, showing higher efficacy against *S. aureus* and *C. albicans*
[50]	Dendê, soy, corn, rice and sunflower oils	PV = 7–1746 mEq O_2_/kg	*S. aureus* (ATCC 25923), *E. coli* (ATCC 25922) and *P. aeruginosa* (ATCC 27853)	The results showed that dendê oil has a better antimicrobial activity with an extraordinarily low MIC (4.75; 2.37 and 1.9 mg/mL) for the three microorganisms studied
[58]	Virgin and pomace olive oils	13.03–1067.23 mEq O_2_/kg	*E. coli* (STCC 45)* P. aeruginosa* (STCC 109)* S. aureus* (STCC 239)	Antimicrobial activity against *S. aureus* was stronger than against *E. coli* and *P. aeruginosa*, with the highest inhibition diameters (18.97 ± 1.46 mm) observed after 180 days of storage (4 °C).
[78]	Olive oil (OO)	PV = 862–2506 mEq O_2_/kg	*S. aureus* (ATCC 6538), *E. coli* (ATCC 10536), *Pseudomonas aeruginosa* (ATCC 27853), and *Bacillus subtilis* (ATCC 6633)	0.95 mg/mL MIC values for both oils (SO and OO) for all the tested strains except against *P. aeruginosa*, where sunflower oil at low peroxide value had better antimicrobial activity.
	Sunflower oil (SO)	PV = 735–2439 mEq O_2_/kg		
[79]	Sunflower oil	Not available	*S. aureus* and *E. coli* from mares with endometritis	*E. coli*: No antimicrobial activity *S. aureus*: 512 µg/mL (MIC)
[80]	Olive oil (O)	PV = 703.7 mEq O_2_/kg	*E. coli* (ATCC 700609)	O: 4.5 mg/mL (MIC) V: 4.5 mg/mL (MIC)
	Venadillo oil (V)	PV = 892.12 mEq O_2_/kg	*S. aureus* (ATCC 2921)	O: 2.5 mg/mL (MIC) V: 1.5 mg/mL (MIC)
[81]	Olive oil (O)	PV = 3110 mEq O_2_/kg	*Candida albicans*, *E. faecalis*, *E. coli*, *S. aureus*, *P. aeruginosa* and *K. pneumoniae*	*C. albicans:* Ø > 20 mm in both oils *E. coli* and *E. faecalis*: Ø > 7 mm in both oils *S. aureus:* Ø > 10 mm in sunflower oil
	Sunflower seeds oil (S)	PV = 3520 mEq O_2_/kg		
[82]	Olive oil	PV = 1280 mEq O_2_/kg	*Alternaria alternata* (ITEM 752)	5 mg oil: Ø > 18 mm 10 mg oil: Ø > 22 mm and 20 mg oil: Ø > 28 mm
[83]	Neozone^®^ Sunflower oil	PV = 4000 mEq O_2_/kg	*E. coli*, *S. aureus*, *S. zoopidemicus*, *P. aeruginosa*, *K. pneumoniae*, *C. albicans*	Ozonated distilled water did not show a significant antibacterial effect; whereas both gaseous ozone and ozonated oil showed antimicrobial activity against antibiotic-resistant bacterial and yeast strains
[84]	Sunflower oil	PV = 600 mEq O_2_/kg	*Phytium indiosum*	The MIC range was 7000 to 437.5 mg/mL for the ozonated SO, and the values for non-ozonated SO were higher: 56,000 to 14,000 mg/mL
[85]	Extra virgin olive oil	5 min of ozonation with a 1000 mg/h yield at 20 °C	Total Viable Count (TVC), Lactic Acid Bacteria (LAB), *Enterobacteriaceae* (EB) and Coliforms (CF)	Extended shelf life from 3 to 15 days
[86]	Sunflower oil	PV = 150, 300 and 600 mEq O_2_/kg	*E. coli* isolates from the intestinal microbiota of healthy rats	OSO 150 (goat milk): 14.3 vs. 9.08 log^10^ CFU/mL OSO 300 (sheep milk): 14.3 vs. 9 log^10^ CFU/mL OSO 600 (cow milk): 20.67 vs. 2.3 log^10^ CFU/mL
[87]	Sunflower seed oil	PV = 91.13 mEq O_2_/kg	*P. aeruginosa* (isolated from mares)	The conventional form of the oil was active against the strains, whereas the ozonated oil was not. Both oils significantly decreased the pharmacological activity of the drugs.
[88]	Oleic acid	4 h of ozonation with a 130 g/m^3^ yield	Prokaryotic and eukaryotic microbiome from the grapevine’s carpoplane	*Acetobacter aceti*, *Pediococcus* sp. and *S. cerevisiae* showed the highest sensitivity

**Table 2:** Summary of the therapeutic properties observed in several ozonated vegetable oils, the peroxide index, the experimental model conditions (in vitro, in vivo) used and their cytotoxicity.

References	Type of Oil	Peroxide Index (mmol/kg)	Model	Toxicity Studies	Therapeutic Properties
[18]	Olive oil and refined olive oil	258.64 and 922.59 for olive oil and 0.91 for refined oil	In vitro model: normal (HaCaT, LLC-PK1) and cancer cell lines (Caco-2, HeLa).	Not cytotoxic up to 625 µg/mL in all tested cell lines.	Antimicrobial (*E. coli*, *S. aureus*, *C. albicans*, *A. brasiliensis*).
[94]	Sunflower	650	In vivo model: rats	No death or malformations.	Antioxidant activity Gastroprotective
[95]	Sunflower	783.4	In vitro model: RAW264.7 and BV-2 cell lines.	No cytotoxicity maintaining 61.9% viability in RAW264.7 and enhanced multiplication on BV-2 cells.	Antioxidant activity Cellular and embryonic protection: Antimicrobial activity
			In vivo model: Zebrafish embryos.	No death or malformations.	
[96]	Ozonated krill oil	Not specified	In vitro model: RAW 264.7 murine macrophages.	No cytotoxicity observed up to 100 μg/mL; cytotoxicity appeared at 200 μg/mL.	Anti-inflammatory
[97]	Sunflower	783.4	In vivo model: adult zebrafish.	Protective effects against CML-induced toxicity, including liver, nervous system, and caudal fin regeneration.	Anti-inflammatory Tissue regeneration Hepatoprotective Neuroprotective Prevention of dyslipidemia
[98]	Sunflower	783.4	In vivo model: zebrafish embryos.	Protective effects against CML-induced toxicity with high survival (61%).	Anti-inflammatory Antioxidant Hepatoprotective Prevention of dyslipidemia
[99]	Sunflower	783.4	In vivo model: adult zebrafish.	No adverse effects in vital organs (liver, kidneys, testes, ovaries).	Anti-aging Organ-protective Prevention of dyslipidemia
[100]	Sunflower	783.4	In vivo models: rats and zebrafish embryos and adults.	No toxicity or embryonic mortality. Protective effect against hepatic and neuronal toxicity caused by CML.	Antioxidant Anti-inflammatory Prevention of dyslipidemia
[101]	Olive oil	Not specified	In vivo model: obese Zucker rats.	No direct cytotoxicity reported.	Attenuation of hepatic steatosis, reduction in hepatic triglycerides, and suppression of inflammatory factors.
[102]	Olive oil	Not specified	In vivo model: obese db/db mice and healthy C57BL/6J mice.	No direct cytotoxicity reported.	Reduces hepatic steatosis Decreases insulin Downregulates lipogenic and inflammatory gene expression

## Data Availability

No new data were created or analyzed in this study. Data sharing is not applicable to this article.

## Supplementary Materials

The following supporting information can be downloaded at: https://www.mdpi.com/article/10.3390/foods15111850/s1, Supplementary data SI.
