# Supplementary material for: Exploring Ozonated Vegetable Oils as Antimicrobial and Functional Agents in Food Systems: A Systematic Narrative Review
Source: Foods. 2026 May 23;15(11):1850. doi: 10.3390/foods15111850 (PMC13257085; doi:10.3390/foods15111850)
Supplement: Supplementary file 1 [file foods-15-01850-s001.zip › foods-4298420-supplementary.pdf]

Supplementary data SI

Taking into account the Topics available in Scopus and Web of Science the research areas were grouped into **11 clusters** as follows:

1. Food Science and Technology

Food Science Technology

2. Agricultural Sciences

Agriculture Dairy Animal Science
Agronomy
Agricultural and Biological Sciences

3. Environmental and Earth Sciences

Environmental Sciences
Engineering Environmental
Environmental Science
Earth and Planetary Sciences

4. Chemistry and Chemical Engineering

Chemistry Multidisciplinary
Chemistry Medicinal
Chemistry Applied
Chemistry
Chemical Engineering

5. Biological Sciences

Biochemistry Molecular Biology
Biochemistry, Genetics and Molecular Biology
Cell Biology
Biology
Inmunology

6. Microbiology

Microbiology
Biotechnology Applied Microbiology
Immunology and Microbiology

7. Medicine and Health Sciences

Medicine General Internal
Medicine Research Experimental
Surgery
Dermatology
Ophthalmology
Nursing
Health Professions

Dentistry Oral Surgery Medicine
Dentistry

8. Pharmacology

Pharmacology Pharmacy
Pharmacology, Toxicology and Pharmaceutics
Integrative Complementary Medicine

9. Veterinary Sciences

Veterinary Sciences
Veterinary

10. Engineering, Energy and Materials

Engineering
Agricultural Engineering
Energy Fuels
Energy
Materials Science
Physics and Astronomy

11. Others

Computer Science
Business, Management and Accounting
Social Sciences
Multidisciplinary
